# A model and simulation of the emotional contagion of netizens in the process of rumor refutation

**DOI:** 10.1038/s41598-019-50770-4

**Published:** 2019-10-02

**Authors:** Runxi Zeng, Di Zhu

**Affiliations:** Center for Communication and Social Development, School of Journalism & Communication, Chongqing University, Chongqing, 401331 China

**Keywords:** Computational science, Complex networks, Nonlinear phenomena, Statistical physics

## Abstract

The emotional contagion of netizens is an important factor that accelerates the spread of rumors, and it is also key to the effectiveness of rumor refutation. Based on the existing emotional model, we improved the method for calculating the emotional value and the transformation rules to simulate how the infection transforms individual emotion to group emotion during rumor refutation. The results show that the cycle and trend of netizen emotional change vary by period, but the final distribution structure presents a relatively stable state. The factors that affect the emotional changes of netizens are mainly objective and subjective aspects, both of which can promote the evolution of emotional contagion. The objective aspect depends on the speed and credibility of the rumor, and the subjective aspect depends on the degree of intimacy between netizens. After rumor refutation, emotions generally change from negative emotions to positive or immune emotions.

## Introduction

Emotional contagion means that in daily life, emotions such as happiness, anger and sadness can be “transmitted”, like an infection, from one person to another in a short time^1^. Each person is an individual emotional sensor, and emotional contagion can not only change people’s emotions but also act as a signal to affect the emotions of others^2^. Stieglitz *et al*.^3^ argue that emotions are an important potential driver of information diffusion in social media environments, especially when netizens share information, as the driving effect is more significant in this case. In a network society, the stronger the emotional experience of individual netizens, the stronger the diffusion of social media. Relevant research shows that emotions with strong emotional polarity, such as anger, easily spread^4,5^. The emotional contagion hypothesis explains how emotions are transmitted and diffused during the dialogue between communicators and receivers and eventually become large-scale group emotions^6,7^.

The contagion path of group emotion can often be followed, which provides a research perspective for explaining the emotional diffusion path of network group behavior intention in uncertain information scenarios. It has been pointed out that the subject of group emotion must be an individual who regards themselves as a member of the group^8^, and the subjective identity of the individual within the group is the correlative index of the group emotion, which determines the individual’s emotional response to a certain event^9^. This collective phenomenon has also been attributed to a large number of scattered microemotional interactions stemming from macroresults^10^. As a network structure with irregular and developing characteristics, the emotional contagion process is abstracted as the connection between network nodes and the change in state^11,12^.

Vosoughi *et al*.^1^ define online rumors as information that has been verified as true or false that spreads or diffuses through a network. Emotional contagion affects the behaviors of both individuals and groups. That is, rumors are inherently social and involve the sharing of claims between people. Online rumors themselves are a distorted reflection and alternative expression of various social conflicts, public needs and social anxieties^13,14^. People experience all kinds of emotions when faced with rumors; in particular, contagion of negative emotions such as panic can have devastating consequences for humans in emergency situations^15^. Na *et al*.^16^ proposed a novel mechanism for explaining rumor acceptance, arguing that the congruence between one’s emotional state and the emotion induced by a rumor leads people to believe the rumor.

The relevant management departments or subjects involved are tasked with refuting rumors to try to eliminate the rumors through clarification or explanation via official channels or mass media and other communication channels^17^. After accepting the rumor “contagion”, the audience produces a series of emotional reactions, which then affects rumor refutation^18^. To achieve a strong rumor refutation effect, the emotion of netizens cannot be ignored. Emotional contagion has long been considered to be an automatic transition of emotional states between people, similar to the spread of disease. However, Wrobel *et al*.^19^ challenged this view, arguing that, unlike disease, emotional contagion is controlled by social factors and is not transmitted blindly.

According to previous studies, pirates and netizens can have an impact on the process of emotional contagion and the effect of rumor refutation. Anti-rumor credibility^20–22^ and speed of response^23,24^ can weaken the threat of rumors, ease people’s panic and anxiety, and effectively control the deterioration process of rumor emergencies. Ordinary netizens, as the main group promoting the dissemination of information and emotion, the self-judgment ability of individual nodes^16^, the subjective willingness to spread^7,25^ and the intimacy of adjacent nodes^26^, are the personal factors that affect the acceptance and dissemination of emotions. Therefore, regarding the factors of emotional contagion during rumor refutation, this study will consider the credibility of subject *a*_*s*_, the speed of clarifying rumors *v*_*s*_, the ability to judge *d*_*r*_, the ability to express emotions *w*_*r*_, and the willingness to spread a rumor *b*_*r*_ (Table 1).

**Table 1:** The influencing factors of netizens’ emotions can be divided into objective and subjective aspects. The objective factors consider credibility, such as the antirumor subject *a*_*s*_, and the speed of clarifying rumors *v*_*s*_, and the subjective factors consider the individual subjective judgment ability *d*_*r*_, the emotional expression ability *w*_*r*_, and the willingness to spread a rumor *b*_*r*_.

Parameter symbol	Meaning
*a*_*s*_ (0 ≤ *a*_*s*_ ≤ 1)	Credibility: indicating the credibility of the subject, such as government officials, experts and scholars, or opinion leaders.
*v*_*s*_ (0 ≤ *v*_*s*_ ≤ 1)	The speed of refuting the rumor: the speed of the subject’s refutation of the rumor.
*d*_*r*_ (0 ≤ *d*_*r*_ ≤ 1)	Subjective judgment ability: among individual netizens, due to individual differences, the degree of rationality will also differ.
*w*_*r*_ (0 ≤ *w*_*r*_ ≤ 1)	Expression ability: the ability of individual netizens to communicate and express themselves.
*b*_*r*_ (0 ≤ *b*_*r*_ ≤ 1)	Willingness to spread a rumor: the subjective willingness of individual netizens to spread a rumor.

Some studies suggest that research on rumors and rumor refutation should emphasize the “social” perspective of social psychology and analyze methods of controlling rumors at the personal and social levels^27^. Previous studies have focused on how the network structure between netizens contributes to the viral spread of rumors^28^ or the emotional state of netizens during rumor spreading^16^. However, few studies have focused on the relationship between psychology and social network structure during rumor dismissal. In addition, although emotional contagion has been studied from a computational point of view^29^, most of the studies consider binary emotional states, and neutral individuals have not yet been considered^30–32^.

The purpose of this paper is to reveal the changing process and evolution of netizen emotions during rumor refutation and to explore how individual emotions form group emotions under the influence of emotional contagion and thus affect the overall momentum and trend of public opinion. To some extent, emotional contagion is similar to disease transmission, and epidemiological models, such as the SIR model, are widely used^33,34^. According to the Zhao *et al*.^32^ contagion model, we improve the classification of emotions and propose to adapt an emotional multiagent state switching model to the process of rumor refutation. The aim is to analyze the process of an individual emotional state, the overall trend of emotional change and the influencing factors. In the specific research process, this study uses Python and Netlogo tools to compile simulation experiments. Multiple experimental scenarios are simulated based on the value of the parameter variables (Table 2), including network relationship structure, time period, speed of clarifying a rumor and credibility. According to the results of each experiment, we can infer the relationship between the parameter variables and emotional contagion.

**Table 2:** The description of parameters of emotional contagion.

Parameter symbol	Meaning
*N*	Network size; *s* + *r* + *m* = *N*
*s*, *r*, *m*	Emotional spreader, Emotional infected, Emotional immunized
*λ*	Emotional contagion threshold, 0 ≤ *λ* ≤ 1
*E*_*s*_(*t*)	Emotional value of *s* at time *t*, 0 ≤ *E*_*s*_(*t*) ≤ 1
*E*_*r*_(*t*)	Emotional value of *r* at time *t*, 0 ≤ *E*_*r*_(*t*) ≤ 1
*E*_*m*_(*t*)	Emotional value when *m* at time *t*, 0 ≤ *E*_*m*_(*t*) ≤ 1
*f*_*s*_	The probability of *m* being converted to *s*, 0 ≤ *f*_*s*_ ≤ 1
*f*_*r*_	The probability of *m* being converted to *r*, 0 ≤ *f*_*r*_ ≤ 1
*R*_*sr*_ (*t*)	The influence of *s* to *r* on emotion at time *t*, 0 ≤ *R*_*sr*_ ≤ 1

## Simulation Results

Through four groups of experiments, this study set up experimental parameters and simulation analyses of the model. Experiment 1 analyzed the distribution of and variation in different types of emotional subjects in different time stages by iterating the contagion cycle of emotional subjects. Experiment 2 controlled the values of *w*_*r*_, *b*_*r*_, and *v*_*s*_ (Table 1) in *R*_*sr*_ (Table 2) to observe the influence of different degrees of network intimacy on the changes in levels of competition between emotional subjects and the process of changing the state of infected subjects. Experiment 3 analyzed the influence of the speed and credibility of refutation on the emotional value of netizens. Experiment 4 compared the emotional value of netizens before and after rumor refutation. After a series of artificial interventions, the change in the emotional value of the rumor, that is, the merits and demerits of the final rumor-refuting effect, is analyzed.

### Time period of emotional contagion

**Figure 1:**
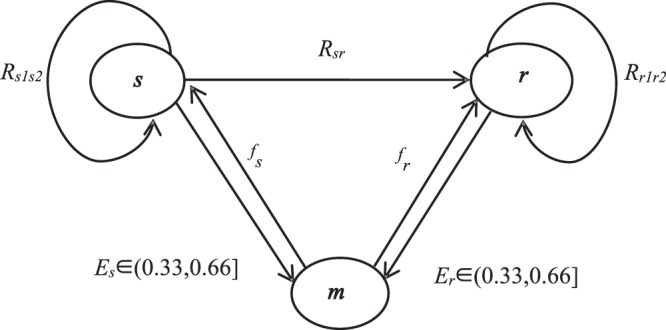
Conversion diagram of the emotional subject relationship. The conversion between *s* and *r* depends on the degree of intimacy between them, and the conversion between *s* and *m* and *r* and *m* depends on the probability of breaking through the emotional critical point.

**Figure 2:**
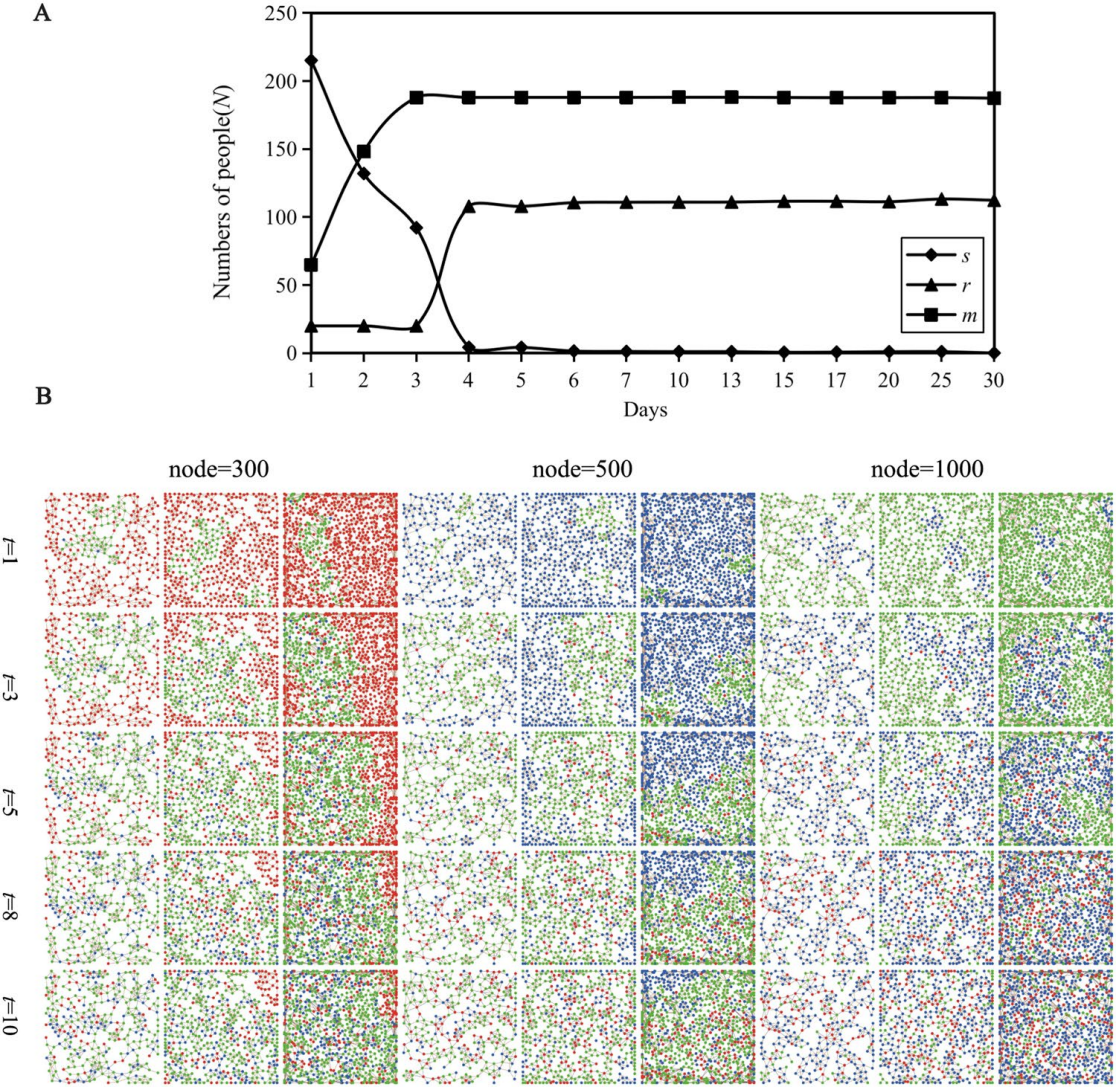
The time distribution of the subject of emotional contagion. (**A**) The overall increasing and decreasing trend of *s*, *r* and *m*. (**B**) Distribution of the emotional subjects at *t* = 1, 3, 5, 8 and 10 days. The red subject emotional state is negative, *Es*(0) = [0.00, 0.33], the blue subject emotional state is immune, *Es* (0) = (0.33, 0.66), and the green subject emotional state is positive, *Es* (0) = (0.66, 1.00].

Emotional contagion is a highly dynamic social network in which individuals constantly rebuild or break old social connections^35^. Thus, to explore the expansion process of the dynamic network, the emotional state and emotional value of each subject were recorded at different times. The time period of the iterative model (*t* = 30 days) is used to observe how the emotional subject changes with the time of refuting the rumor under the condition of emotional state switching rules (Fig. 1). *s* was the most active in the early stage of rumor refutation and gradually disappeared after promoting the transformation of netizens into *r* and *m* (Table 2). Regarding *r*, in the first three days, the number of people did not change significantly, but the number rapidly increased to a fixed value after three days, and the stable time was roughly in sync with *m* (Fig. 2A). The number of emotional subjects was basically stable after 4 days. It can be speculated that in the days before a rumor, netizens are the most vulnerable to infection, and the number of *r* also reached a peak at this time.

Rumors spread through social media, with a large number of users spontaneously discussing, questioning, complementing, correcting and disseminating relevant authoritative information; therefore, the influence of rumors gradually declines and eventually loses its vitality^36^. Figure 2 shows that if the initial emotion of *s* is mainly a negative emotion (*Es*(0) = [0.00, 0.33]), then the negative emotion remains for a long time and dissipates slowly; if the initial emotion of *s* is mainly a neutral (*Es*(0) = (0.33, 0.66]) or positive emotion (*Es*(0) = (0.66, 1.00]), there will be less negative emotion while refuting the rumors. However, common to the three kinds of emotions, at the end of the rumor refutation, most netizens’ emotions are neutral and positive. Another interesting finding is that when the initial emotion of *s* is negative and neutral, more individuals tend to be positive, while when the initial emotion of *s* is positive, more individuals tend to be immune. Finally, the convergence time of the large-scale network contagion process is long, and its final stabilization effect is relatively poor^37^.

This point is when most people’s emotions change to immune or positive states. Netizen emotions mainly appear at the beginning of rumor refutation (Fig. 2B) and peak in the first two or three days, but as the time over which the rumor is released increases, the negative emotion gradually decreases after a week, finally dissipates, and tends to reach a stable value. The experimental results show that all subjects may be in different states, but eventually, they will achieve dynamic equilibrium and tend to be stable^38^. Ultimately, netizen emotions are mostly transformed into immune or positive emotions, and very few remain in a negative state.

### The influence of netizen relationship structure on emotional contagion

The value of *R*_*sr*_ is simulated and analyzed, and the results show that the higher the value of *R*_*sr*_, the closer the relationship between *r* and *s*, and the better the propagation effect. When the time variable was controlled, the influence of *R*_*sr*_ on emotional contagion was observed. The emotions of netizens returned to rational or immune status after a week (Fig. 2A). Therefore, in experiment 2, *t* was set conservatively to 10 days to ensure that netizens’ emotions reached a rational state during the period.

**Figure 3:**
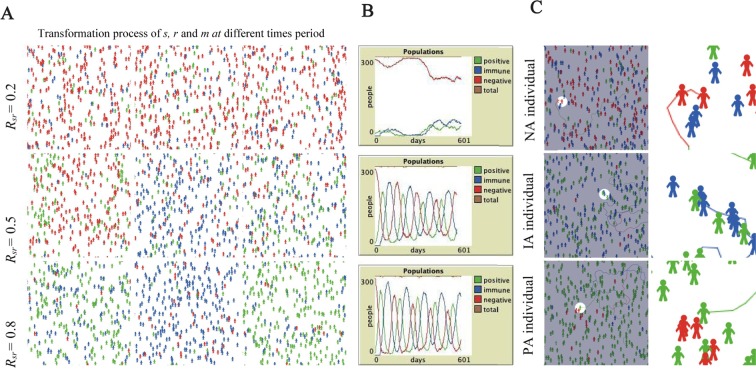
The influence of subjective factors on emotional contagion. (**A**) When *R*_*sr*_ = 0.2, 0.5 and 0.8, regarding the contagion distribution of the subject of emotion, the red individual was negative, the blue individual was immune, and the green individual was positive. (**B**) Under the above three conditions, the numbers of *s*, *r* and *m* change. (**C**) The emotional transmission of individuals in the negative (NA), immune (IA) and positive (PA) states to the surrounding individuals.

The influence of the time factor on the emotional contagion of netizens was excluded. After controlling for the time variable, the affinities of individual netizens were observed. As Fig. 3A shows, *r* is unlikely to be infected by *s*, and the possibility that the infection will cross the interaction space is small. Most netizens are in the initial negative emotional state (*R*_*sr*_ = 0.2, *t* = 10 days). *s* has a certain influence on *r*, there are overlapping and intersecting parts in the interaction space, and the number of *r* and *m* increases noticeably (*R*_*sr*_ = 0.5, *t* = 10 days). When the emotions of most netizens tend to be positive or immune, the interaction of *s*, *r* and *m* is obvious (Fig. 3B) and the effect is the strongest (*R*_*sr*_ = 0.8, *t* = 10 days).

In an individual-centered network society, people tend to connect with people who are similar to themselves^39^. After forming closely related groups, the high-density network society can help people quickly identify rumors through collective intelligence under the influence of multisource interaction and emotional contagion^33,40^. This kind of “contagion model” based on kinship and alienation is not a one-way model, but a mutual feedback transmission, which is conducive to emotional aggregation during the formation of a climate of opinion, leading to an upward trend of group emotions^41^. This model even circulates and amplifies in the media context, resulting in “emotional flow waterfalls”^42^.

When most individuals are in a negative emotion, the person closest to each individual is in the same emotional state (Fig. 3C). It is also possible that, after contact with others and being affected by their emotions, individuals hold a neutral attitude toward rumor refutation or transition to positive emotions and further infect the people around them. In the form of interpersonal transmission, the contagion process of rumor information is accelerated, which ultimately leads to the group emotion achieving a positive or immune state.

### The impact of the speed of rumor refutation and credibility on emotional contagion

**Figure 4:**
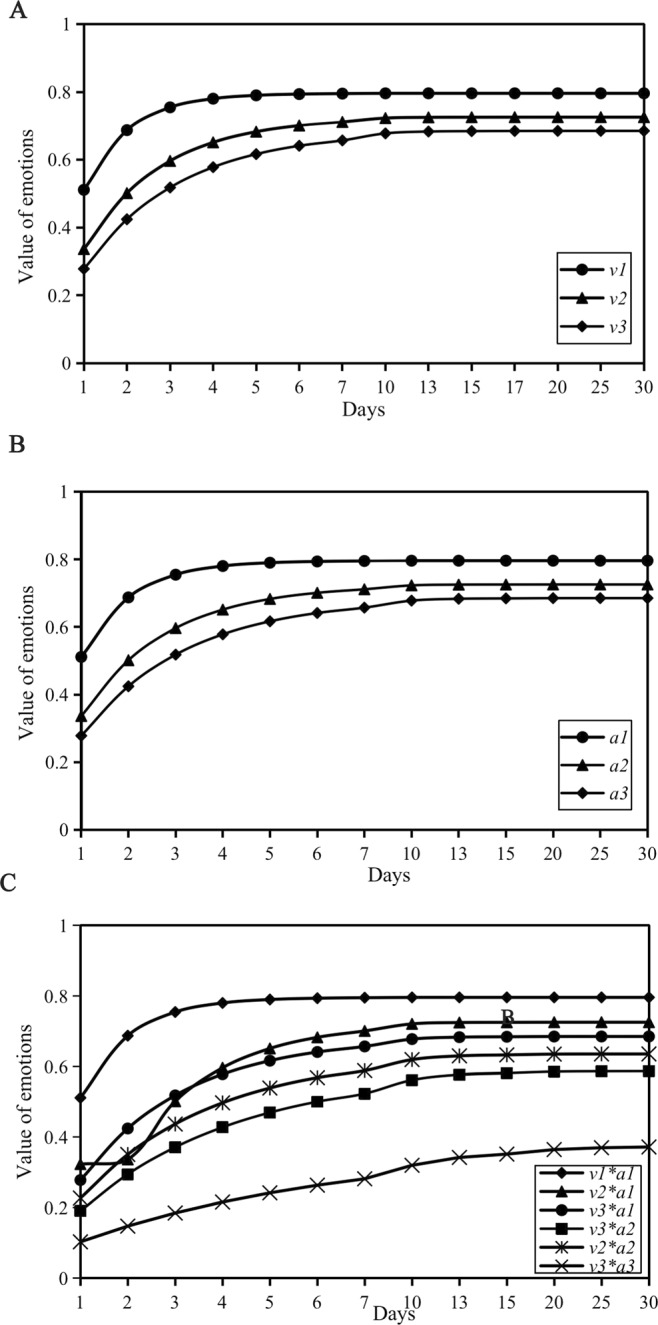
The influence of objective factors on emotional contagion. (**A**) The influence of different *v*-values on the emotional value of netizens. (**B**) The influence of different *a*-values on the emotional value of netizens. (**C**) The influence of different combinations of *v* and *a* on the emotional value of netizens.

*v* and *a* can affect the emotional value of netizens to a certain extent. The greater the values of *v* and *a*, the more likely the netizens’ emotions are to eventually change to positive emotions (Fig. 4A,B). To further explore the impact of the interaction of the two factors on the emotional value of netizens through the permutation and combination of three groups of values of *v* and *a*, six combinations, *v*1 * *a*1*, v*2 * *a*1*, v*3 * *a*1*, v*2 * *a*2*, v*2 * *a*3 and *v*3 * *a*3, are finally obtained, representing combinations of *v* and *a* of different sizes (Fig. 4C). In the combination *v*1 * *a*1, that is, when *v* and *a* are the highest, the effect of refuting the rumor is at its best, and most of the emotional values of netizens are stabilized in the range of positive emotions after 2–3 days (*v* = 0.8, *a* *=* 0.8, *E*_*r*_ = 0.81). However, the combination *v*3 * *a*3 is the worst, and the effect of rumor refutation is not ideal. Almost all netizens were at the intersection of negative and immune emotions (*v* *=* 0.2, *a* *=* 0.2, *E*_*r*_ = 0.68). This finding shows that for the time examined, the faster the speed of refuting the rumor, the higher the credibility and the greater the impact on the emotional value of the netizens.

### Comparison of netizen emotions at the beginning and end of rumor refutation

**Figure 5:**
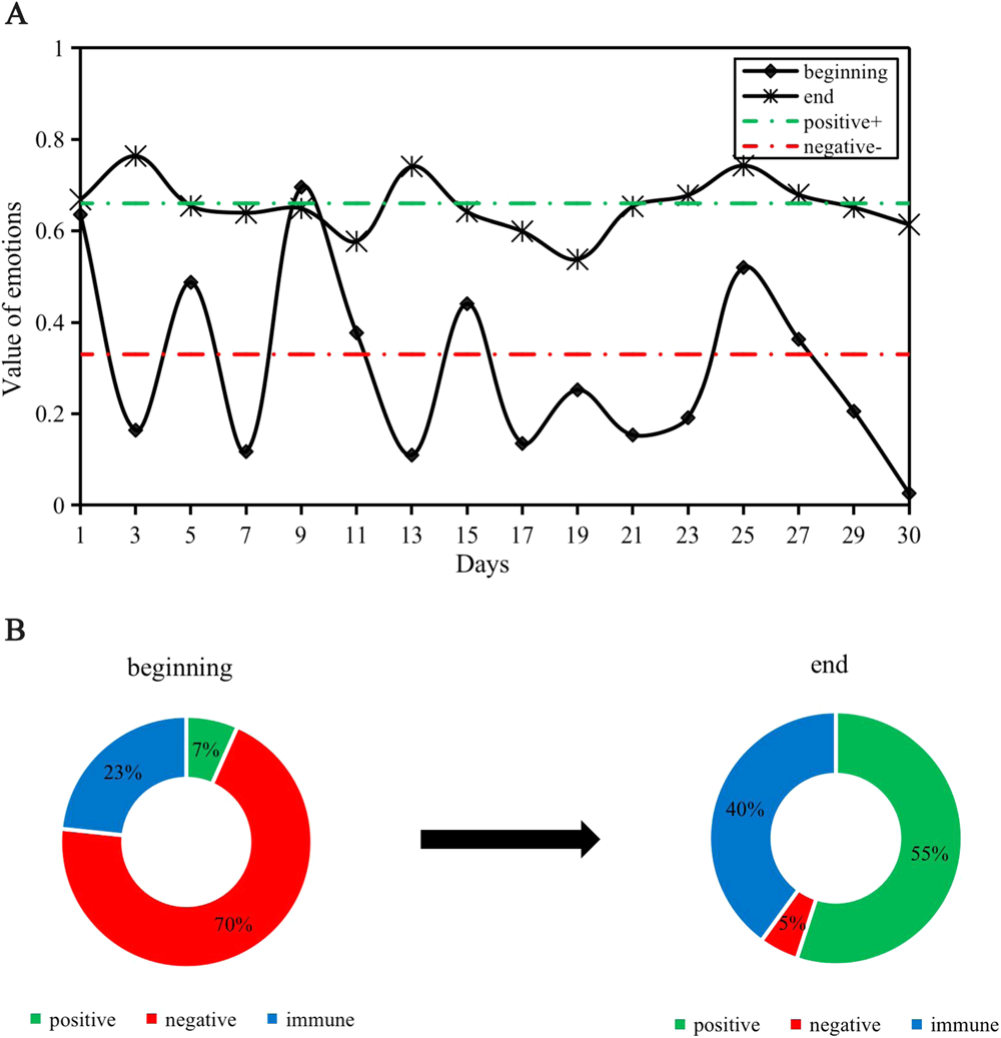
The change in subject emotion at the beginning and end of rumor refutation. (**A**) Comparison of subjective emotional values before and after dismissing the rumor. (**B**) The proportion of subjective emotion before and after dismissing the rumor.

Whether near the beginning or end of the rumor, the emotional value of netizens has repeated high and low peaks (Fig. 5A). Some studies have shown that when cyberspace is limited, that is, the upper limit is low, public opinion information will quickly be saturated at a low value. In contrast, if the attention of netizens is increasing, it will stay at the peak value after rising to a certain maximum level.

This phenomenon is similar to “energy conservation”. Emotional values do not continue to grow infinitely after reaching a certain peak. Instead, they are undulating, fluctuating back and forth between minima and maxima in a negative or positive emotional wave^8^. As a subject’s emotional values fluctuate, the node may be infected by other people’s emotions^11^.

Before and after refuting the rumor, the emotion of netizens fluctuated obviously. Before refuting the rumor, the majority of netizens’ emotional values were lower than 0.66; they were in a negative and immune state, and the emotions of the minority were positive. However, most final emotional values of netizens were in the 0.66–0.87 range, and a few individuals’ emotional values were lower than 0.66 (Fig. 5A). This finding indicates that at the end of rumor refutation, the attitude of most group members toward the information had changed to positive or immune, and negative individuals were rare (Fig. 5B).

Consistent with the results of Zhao *et al*.^32^, this experiment found that negative, highly aroused emotional information can easily get people’s attention, leading to dissemination^43^. Positive emotions such as excitement and happiness transmitted between strangers are negligible, but negative emotions such as anger and sadness can be transmitted among strangers in a large area, gathering and forming an emotional ripple effect^44,45^. This also explains why the effect of refuting rumors is not ideal when the netizens’ negative emotions are strongest, because refuting rumors at that moment will make people more convinced of the original view; this phenomenon is called the “backfire effect”^46^. However, with the convergence of diverse views and emotions and the formation of full discussion or debate, netizens’ rationality gradually returns under the self-purification mechanism of the social platform^23,47^.

## Results Demonstrate

### Data source

On October 28, 2018, the news reported that the Chongqing bus crash was caused by a female driver driving a private car. Many media outlets reported this news, causing some netizens to condemn the female driver. During this period, public opinion chastised the female driver, and accusations flooded the internet. On the evening of October 28th, Chongqing’s official Weibo issued a notice stating that the accident was caused by a bus suddenly crossing the center line while driving, crashing into a private car and then falling into the river. Public opinion began to change, and many netizens apologized to the female driver. On November 2nd, officials officially announced the cause of the bus crash in Chongqing, and public opinion reached its peak on this day. Subsequently, the amount of public opinion gradually showed a downward trend.

The reasons for selecting this example are as follows: first, the incident involves social public concern and sensitive topics and has great social impact. Internet public opinion and the content of the netizens’ comments are conducive to the study of the spread of emotional contagion among netizens. Second, the incident involves anti-rumors and the change process of netizens’ attitude toward female drivers, which fits the induced conditions of emotional transition in the model. Third, the outbreak and fermentation of the event, a series of turning points and discussions caused by the follow-up reports of the major media and the disclosure of the truth were first released on Weibo. The amount of information provided by the Sina Weibo platform in this incident was much higher than that of other media sources. This platform played an important role in exposing and promoting the incident, which helps us to obtain more core views and comments on the platform to analyze its emotional tendencies and emotional infection process. Based on the above facts, the “Chongqing Bus Falling River Event” was selected as the source of information for data acquisition.

### Search condition

Using the advanced search function of Sina Weibo, we selected a 13-day period from 10:00 on October 28, 2018, to 10:00 on November 9, 2018. The keywords “Chongqing Bus Falling Into The River”, “Chongqing Bus Falling Into The River + Female Driver” and “Chongqing Bus Falling Into The River + Female Driver + High Heels” were used as the retrieval keywords. The search condition “Type” was set to select the original popular Weibo, “include” was set to select all, and the retrieval operation was conducted.

### Collection rule

Through the web data collection tool “Octopus”, the following information was collected from the retrieved data: user name, user ID, release time, published content, published URL, forwarding number, number of comments, likes, and other basic information attributes, which were saved to an Excel spreadsheet. In this paper, a total of 24,692 microblog source data were collected.

### Text processing

Due to the arbitrariness of the information release in the context of the Internet and the nonstandardization of the form of information, it was inevitable that some incorrect, unrelated, blank or repetitive data would be retained. Therefore, it was necessary to further clean the data manually. The main operation steps were as follows: manually deleting blank and invalid data, such as the URL string at the beginning of http and words with no actual semantics, and manually deleting unrelated items, such as marketing information, advertising, spam and format words, such as “forwarding Weibo”. Excel was used to remove repeated input data and to delete comments with only pictures or meaningless characters, such as #, and @. Finally, a total of 16,083 valid texts were obtained.

In this paper, the ROST CM text mining software was used to segment the words and frequency of the preprocessed microblog data and to calculate the emotional intensity of the microblog content in the corpus to be analyzed. According to the emotional intensity value, the microblog content was divided into positive microblogging, neutral microblogging and negative microblogging, consistent with the emotional categories of this study. According to the system, 6,389 (39.73%) positive microblogs, 4,679 (29.09%) neutral microblogs and 5,015 (31.18%) negative microblogs were obtained.

### Data analysis

To verify the results of the simulation model, this paper will test the feasibility of our model by combining the four previous experiments with the real case of the Chongqing bus falling into the river.

### Time period of emotional contagion (Experiment 1)

**Figure 6:**
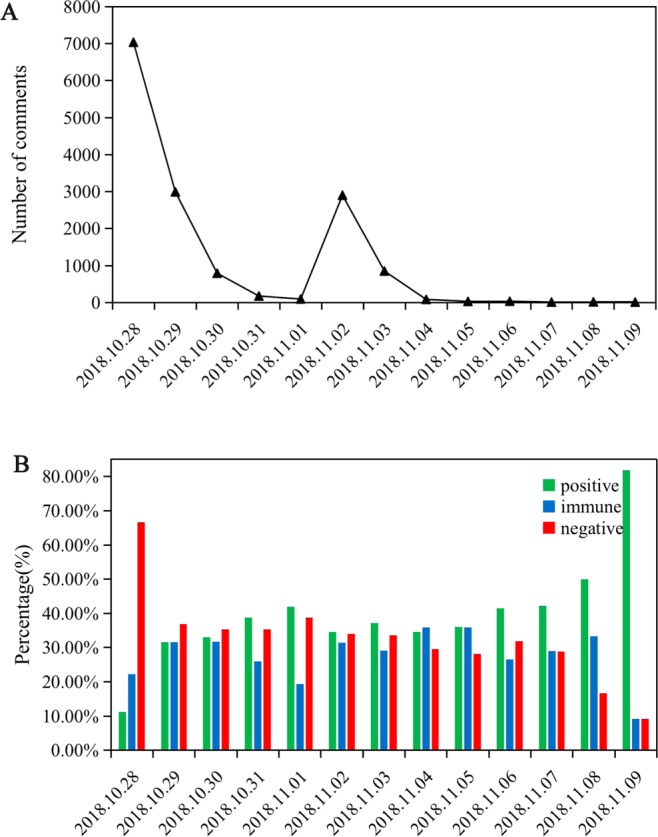
Topic heat index and emotional trend during the process of refuting rumors. **(A)** Topic heat index. **(B)** Emotional tendency of netizens.

Based on the processed research samples, this paper created a topic heat trend about “female drivers of buses falling into the river in Chongqing” (Fig. 6A). On the 28th day, it received the most attention, followed by the 2 days when the truth of the fall into the river was announced, which again aroused discussion among netizens. Consistent with Experiment 1, before refuting the rumor, the emotion of the netizens was mainly negative. After the rumor was refuted, the negative emotion significantly decreased. From November 7th to November 9th, the emotions of most netizens were transformed into positive emotions and neutral emotions, especially on the 9th. The reduction in negative emotions was the lowest (Fig. 6B).

It can be seen that the selected rumor case is not only consistent with the 10-day cycle of Experiment 1 but is also consistent with the emotional tendency distribution and transformation results, which demonstrates that the data of Experiment 1 simulated by the simulation experiment are reasonable.

### The influence of netizens’ relationship structure on emotional contagion (Experiment 2)

The original Experiment 2 compared the effect of refuting rumors by assuming that *s* and *r* had different degrees of closeness. However, it is difficult to accurately quantify the closeness between *s* and *r* in a real environment. Therefore, this paper relies on the number of comments, likes, forwards and fans as the reference index of the relationship between *s* and *r*. We selected media sources that differed from the netizens in terms of closeness and influence. These sources, including the “Phoenix Weekly”, “Daily News” and “People’s Daily”, represented the rumors (*R*_*sr*_ = 0.2, 0.5 and 0.8). The comments of the three media sources were collected, and their effect on refuting the rumor was analyzed.

**Figure 7:**
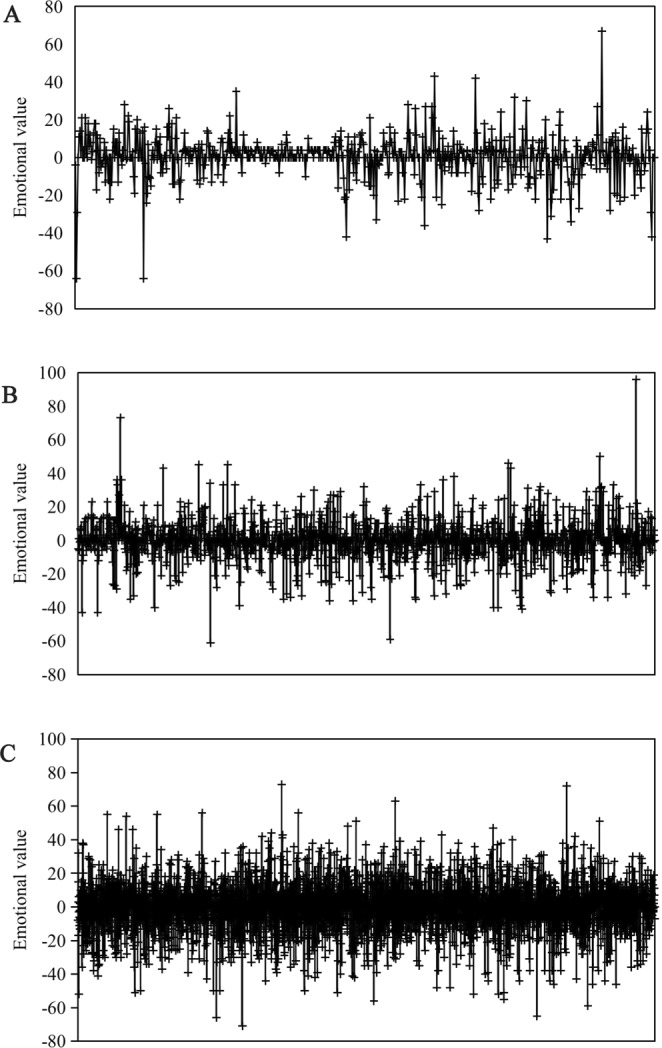
The changing trend in the netizens’ emotional value under the influence of media sources refuting rumors. **(A)** *R*_*sr*_ = 0.2 **(B)** *R*_*sr*_ = 0.5 **(C)** *R*_*sr*_ = 0.8.

The closer the relationship among netizens is, the greater the influence of the main body. In the process of refuting rumors, the greater the probability is that netizens will discuss the rumors, the more obvious the emotional amplitude of netizens is (Fig. 7). Evidence shows that the closer the relationship between *s* and *r* is, the more extensive the discussions are that will be gathered. In this environment, the emotional contact rate between groups is higher and it is easier to form group momentum, which helps to enhance the spread and infection of netizens’ emotions. Thus, the data of Experiment 2 simulated by the simulation experiment are consistent with the actual case.

### The impact of the speed of rumor refutation and credibility on emotional contagion (Experiment 3)

**Figure 8:**
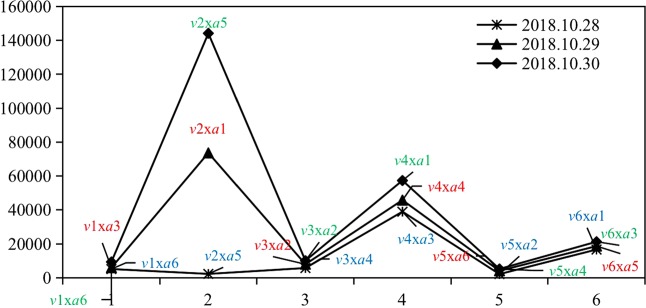
Different effects of refuting rumors produced by different combinations of *v* and *a*.

In this paper, the date and time indicators to refute rumors are represented by *v*, and the attention of bloggers, the number of fans and blog posts are represented by *a*. According to these indicators, *v* and *a* are sorted and combined. The final effect of refuting the rumor is expressed by the forwarding, comments and likes of the content sent by bloggers (Table 3). The results show that, consistent with Experiment 3, the influence of *a* is weaker than that of *v*, and *v* is the key to refuting rumors (Fig. 8).

**Table 3:** Indicators of the speed, credibility and influence of the media in refuting rumors.

Official media	*v*			*a*				influence		
	date	time	*v* valuse	followers	fans	weibo	*a* valuse	forward	comment	like
China Fire	2018.10.28	14:31	*v*11	995	3207416	31271	*a*16	110	2341	2721
Chengdu Commercial Daily	2018.10.28	17:55	*v*12	854	13139957	124385	*a*15	152	961	1194
Sina Video	2018.10.28	18:21	*v*13	1834	13361884	86652	*a*14	851	1767	3149
Phoenix Weekly	2018.10.28	19:00	*v*14	562	18734749	48258	*a*13	4668	11420	22894
Daily News	2018.10.28	23:30	*v*15	1382	64875331	160387	*a*12	370	837	979
People’s Daily	2018.10.28	23:55	*v*16	3034	89496962	100321	*a*11	2012	2520	12140
Sina Video	2018.10.29	11:53	*v*21	1834	13361884	86652	*a*23	53	345	413
Daily News	2018.10.29	11:54	*v*22	1382	64875356	160387	*a*21	9458	15817	46080
Sina News	2018.10.29	13:11	*v*23	1886	18109997	47813	*a*22	151	686	1403
Interface News	2018.10.29	13:20	*v*24	846	9107176	42364	*a*24	492	1431	4903
Driving Manual	2018.10.29	14:50	*v*25	626	1386347	6170	*a*26	577	691	669
Observer Network	2018.10.29	15:16	*v*26	1144	8660534	90009	*a*25	201	694	1149
Burning News	2018.10.30	11:27	*v*31	354	885516	10930	*a*36	306	2473	606
Modern Express	2018.10.30	11:41	*v*32	1319	13000029	105362	*a*35	10659	17827	42024
Daily News	2018.10.30	11:49	*v*33	1382	64875657	160387	*a*32	249	796	1055
People’s Daily	2018.10.30	12:20	*v*34	3034	89496962	100321	*a*31	871	3466	7219
Global Network	2018.10.30	12:51	*v*35	1249	14938709	69793	*a*34	146	497	467
China Daily	2018.10.30	13:24	*v*36	856	42350055	106100	*a*33	145	1052	1377

However, in the process of refuting rumors in reality, the ideal combination of *v*1 * *a*1 is relatively rare. A possible explanation is that more authoritative media sources are more cautious; if released news is not true, the damage incurred will be to the credibility and authority of these sources. Therefore, the fastest way to refute rumors is usually not with the most authoritative mainstream media source. In the same period of time, the authority of high-level media sources achieves relatively better results.

### Comparison of netizens’ emotions at the beginning and end of rumor refutation (Experiment 4)

**Figure 9:**
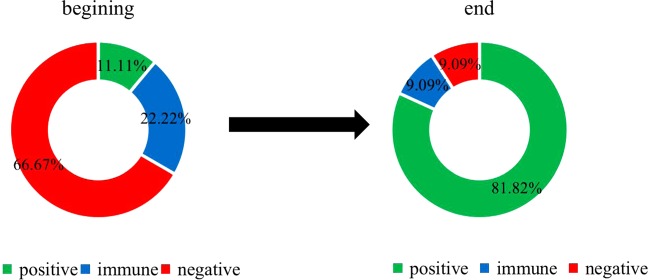
The proportion of subjective emotion before and after dismissing the rumor.

ROST CM software was used to analyze the emotional value from the beginning to the end, and the proportions of positive emotion, neutral emotion and negative emotion were calculated (Fig. 9). In the initial stage of refuting rumors, most of the netizens’ emotions were negative, and the proportion of neutral and positive emotions was low. At the end of the rumor refutation, most netizens’ emotions were transformed into positive emotions and neutral emotions. Therefore, the data of Experiment 4 simulated by the simulation experiment are basically consistent with the real situation.

The results of the four experiments can explain the laws and characteristics of emotional contagion in the real environment to a certain extent. Emotion is the regulator of human behavior and must run well. An effective mode of refuting rumors should be comprehensive and should include not only the information that people can trust the main body refuting rumors on the Internet and that netizens can cultivate rational expressions. This not only provides a new way of thinking about the future of anti-rumor efforts but also lays a foundation for the study of the evolutionary mechanism of group emotions with regard to rumors.

## Conclusion and Discussion

By modeling and analyzing the change in netizen emotions and the rules of subject transformation during rumor refutation, this paper investigates how netizens’ emotions change and the influencing factors involved in the process. Four different experiments were used to simulate the emotional contagion process of netizens. Based on the existing emotional contagion model, the method of calculating the main emotional value and the transformation rule was improved to include the main type of emotion, the contagion scenario, the changing conditions and rules and other elements. The main results of the experiment are reflected in the following three aspects.

First, regarding the emotional change cycle and trend of netizens, the active time and period of *s*, *r* and *m* are not the same, but they all tend toward a stable value in the end (Fig. 2A). Consistent with previous studies, the peak emotion of netizens generally appeared in approximately the first 2–3 days of rumor refutation and then began to decline and dissipate; finally, the distribution structure of the emotional subjects achieved a relatively stable state.

Second, regarding the influencing factors of netizen emotions, the subjective level depends on *R*_*sr*,_ and the objective level depends on *a* and *v*. The results show that the spreading effect of rumor-refuting information is better and the time to refute the rumor is shorter in a “close” social circle than in a “dispersed” social circle (Fig. 7). The benign intervention of an authority to refute a rumor can positively guide a network rumor event and help the sentiment of netizens quickly return to the normal level (Fig. 4)^48,49^. In addition, the results further verify that negative emotions are the main driving emotions of information dissemination. The more attention the information received, the more the widespread dissemination of rumor-refuting information can be promoted, which ultimately makes the emotion of netizens tend toward positive or immune.

Third, the netizens’ emotions change at the beginning and the end of refuting rumors. The main change after rumor refutation is that most negative emotions eventually become immune or positive emotions (Figs 5, 6 and 9). Overall, the evolution of emotions is a gradual process, and there is no sharp transition from one emotional state to another. It is common for negative emotions to become immune emotions or to become immune emotions and then positive emotions through the dynamic transformation process (Fig. 3A,C).

Compared with previous strategies based on the psychology and emotion of the rumor audience, this paper innovatively combined research on rumor refutation and netizen emotion. First, we directly took netizen emotion during rumor refutation as the entry point and explored the characteristics and evolution rules of netizen emotional contagion during this process. Second, the experiment proved the rationality and rigor of the distribution of netizen emotions, the survival cycle of negative emotions and the key factors affecting netizen emotions during rumor refutation, which provided a path for predicting the trend of netizen emotions, knowing public opinions and sorting out netizen emotions. Finally, the experiment also proves that this method is an operable way to suppress the speed and scope of rumor propagation to release rumor-refuting information in time by authorities or organizations, calm netizens and channel negative emotions. Analyzing the influencing factors and interaction rules of people’s emotional contagion in Internet rumors can improve people’s cognition of positive and negative emotions in the information age to guide and alleviate crisis rumors with specific targets.

In future research, we will examine the role of a wider range of emotional contagion factors during rumor refutation and guide rumor crisis events in a rational direction by cultivating the participants’ positive dominant emotions. In addition, we should pay more attention to the phenomenon of the emotional contagion of netizens in different social media to achieve a more comprehensive analysis of the characteristics and laws of emotional evolution.

## Methods

### Model construction

Emotional contagion during the spread of rumors is not a blind, nonselective process but a process deeply rooted in the social background^50^. Therefore, the spread of an emotional state cannot be similar to a viral contagion in terms of a “target hit”^51,52^ but must be decoded according to a certain social background.

Some studies have pointed out that emotional contagion is not a simple imitation and feedback of other people’s automatic emotional mechanisms^53^; rather, in the perception of external emotions combined with a certain social background, self-interpretation, and secondary coding, people form emotional fusion^54^. In the process of infection, in addition to the external noise that weakens the original emotion, the recipient’s own endogenous factors also have a certain impact. For example, personal traits such as skepticism, analytical thinking, cognitive needs and a sense of control are thought to raise doubts about information and its interpretation^55,56^. In addition, DiFonzo^57^ stated that people will choose, interpret and reconstruct information according to their internal value schema, which results in a perceptual bias consistent with that schema, making it easy for people to believe rumors consistent with their own beliefs and values. Thus, individual emotions cannot be unconditionally transmitted to others, and this study suggests that emotional contagion transmission during rumor refutation has its own operating procedures.

In a long-term uncertain environment, the standard of judging the credibility of rumors and anti-rumor information will also be reduced. Especially when the credibility of the government is insufficient and the public generally lacks relevant background knowledge, rumors are easier to spread^58^. Experimental studies have found that people trust rumors because of the reliability of the source, and counter information sent by the reliable source is also acceptable^59,60^. At the same time, in practice, to control the spread of rumors, besides paying attention to the reliability of the rumor source, government departments should also release real information in time to curb the spread of rumors. If the majority of netizens are allowed to discuss freely, the longer the time, the more prevalent negative emotions such as anger and resentment, which is not conducive to the good development of the situation^61^.

The emotions expressed by individuals in the process of refuting rumors can be regarded as feedback or new information to others. The type and degree of emotion depends on the individual. We have proved the following: first, individuals have their own subjective judgment of other people’s emotions, choosing whether they want to be infected by them or withdraw from the process of spreading a rumor^34,62^; second, regarding the willingness to disseminate, when information flows to a certain audience, whether the audience wants to continue disseminating emotions depends on the internal tendency of the audience; third, the extent to which individuals express their own primary emotions involves whether they discuss them fully or have reservations^31,40^; finally, the intimacy of individuals with other nodes in the network is an important factor for determining the scope and degree of contagion^61,63^. The closer the emotional relationship between the nodes and the larger the number of nearest neighbors, the stronger the final intensity of the group emotion and the shorter the time required for emotional emergence^33,64^.

In summary, it can be concluded that credibility (*a*_*s*_), the speed of refuting the rumor (*v*_*s*_), subjective judgment ability (*d*_*r*_), expressive ability (*w*_*r*_), and willingness to spread a rumor (*b*_*r*_) are the key factors in the process of refuting rumors. Most of the research results of information dissemination models are based on SIR virus infection models. Because the spread of information in the network is consistent with the principle of disease transmission in the population, the disease transmission model takes into account that infected individuals can infect any susceptible individual. The principle of information dissemination in the network suggests that netizens who know the information in the network can spread this information to netizens who do not know the information. The complex population is divided into the spreader, *s*; the infected, *r*; and the immunized, *m*^65^.

#### **Definition 1**.

A spreader node is a node that knows the information and has its own emotions.

#### **Definition 2**.

An infected node is a node that has been infected by emotions and is able to spread them.

#### **Definition 3**.

An immunized node is a node that has lost interest in the information but can be activated.

Currently, most existing rumors and emotional contagion models are based on different social platforms, scenarios or events and introduce new node states, thus expanding new communication models, such as SHIR^34^, SLRS^15^, CA-SIRS^33^ and URBD^62^ models. However, the unique characteristics of nodes are rarely considered. In recent years, some studies have begun to pay more attention to the impact of node attributes on the spread of rumors, such as the weight value of nodes^14^. In real social networks, the spread of rumors or emotions is not only related to the node state and the proportion of transition^11^ but also to the individual’s inner emotional activity as the key factor to determine its transformation. Moreover, emotions affect the individual’s daily behavior as well as the information dissemination behavior of individuals.

In view of this, this paper is based on the SIR model, comprehensively considers the characteristics and influencing factors of group emotional contagion in a rumor event, and proposes a modified version of the SRM group emotional contagion model. The model improves the simple transformation and one-way transmission mechanism of the original model and considers the impact of the government and netizens on group emotional contagion in online rumors. In addition, it highlights the infection between individual emotions not only by other individuals in the environment but also by the credibility and speed of rumors and provides a more rational framework for the spread of rumors. The specific conversion rules between them are shown in Fig. 1.

This study assumes that there are *N* individuals, each of whom is in one of the three states. We consider a closed and mixed population consisting of *N* individuals as a social network in which individuals can be represented by vertexes and contacts between people can be represented by edges. We describe the number of spreader nodes, infected nodes, immunized nodes and potential spreaders at time t by *s, r*, and *m*, respectively (obviously, *s* *+* *r* *+* *m* *=* *N*).

If one individual is *s* and another is *m*, then a certain infection probability exists between the two; that is, there is a certain influence relation, *R*_*sr*_, between *s* and *r*. This relation denotes the probability that *s* will infect *r* with its own emotional value. Although *m* is immunized, it can be transformed into *s* or *r* at any time. The probability of *m* transforming into *s* is *f*_*s*_ (0 ≤ *f*_*s*_ ≤ 1), and the probability of *m* transforming into *r* is *f*_*r*_ (0 ≤ *f*_*r*_ ≤ 1). The related parameters of the emotional subject are shown in Table 2.

In this study, we focus on how anti-rumors influence the emotion change of each individual with a different awareness and other factors in every step, leading to a final consensus. In this model, we consider the spread of a single piece of information that initially appears on a single node, and the rumor-spreading process is synchronized as in most previous work for simplicity^66^. In other words, the real world scenario is simplified, with netizens acting at different speeds, it occurs at discrete points over time and each time step, and each node contacts its neighbor to exchange information^66^. On this basis, a mathematical construction is built as follows:

1. Few people have stable emotions at the beginning of refuting rumors, and individuals can change their emotional state at any point in time.
2. Whether an individual accepts a message’s emotion is determined by many factors, as mentioned. Such factors make up the transition probability.
3. Different emotions have different parameter values. The value of each parameter is not fixed. When the individual emotion is in the range [0.00, 0.33], then the emotion is negative; when the emotional value is in the range (0.33, 0.66], then the emotion is immune; and when the emotional value is in the range (0.66, 1.00], then the emotion is positive. When the intensity of an individual’s emotion exceeds a certain threshold, then his or her emotional state has changed.
4. In the early stage of refuting rumors, only s carried emotions. At the very beginning, at least one type of active individual needs to exist in a certain state; thus, that the emotional contagion and conversion process is initiated^35,63^.

*R*_*sr*_ mainly depends on the subjective factor of *r* and the objective factor of *s* (Table 1), both of which affect the probability value of emotional transmission. *R*_*sr*_ should be based on the characteristics of *s* and *r*, with *s* mainly considering *a* and *v*. and *r* is mainly considered from the aspects of the *w*_*r*_ and *b*_*r*_ of individual emotions. The equation is calculated as follows:

1 $${R}_{sr}(t)={w}_{r}(t)\ast {b}_{r}(t)\ast {a}_{s}(t)\ast {v}_{s}(t)$$

Physical and emotional contagion is a social phenomenon. The emotions of group members can be absorbed by other group members, and they can be amplified and superimposed to greatly exceed the original emotional level of the group members^8^. Thus, suppose *E*_*r*_ is the sum of the influence of one’s own emotion and *s* on *r* emotion at *t* + 1 moment; then, *r* can be affected only by *s* at any moment. The equation is as follows:

2 $${E}_{ri}(t+1)={d}_{ri}(t)\ast {E}_{ri}(t)+[{E}_{si}(t)-{E}_{ri}(t)]\ast {R}_{sri}(t)$$

### Model scenario hypothesis

This study mainly considers the contact scenes between *s* and *s*, *s* and *r*, *r* and *r*, *m* and *s* or *r* during rumor refutation. The main scenario is as follows:

Scenario one: *s* and *s*

3 $${E}_{s1i}(t+1)={d}_{s1i}\ast {E}_{s1i}(t)+[{E}_{s1i}(t)-{E}_{s2i}(t)]\ast {R}_{s1s2}(t)$$

4 $${E}_{s2i}(t+1)={d}_{s2i}\ast {E}_{s2i}(t)+[{E}_{s2i}(t)-{E}_{s1i}(t)]\ast {R}_{s2s1}(t)$$

When both sides are *s*, the emotional value at *t* + 1 is the sum of *s* emotions and the “absorbed” emotions at *t* time.

Scenario two: *s* and *r*

5 $${E}_{ri}(t+1)={d}_{ri}(t)\ast {E}_{ri}(t)+[{E}_{si}(t)-{E}_{ri}(t)]\ast {R}_{sri}(t)$$

6 $${E}_{si}(t+1)={E}_{si}(t)$$

where *d*_*ri*_ denotes *r*’s subjective judgment of *r*’s own emotions at time *t*, and the value is close to 1 in the interval [0–1], indicating that *r* is firm to its own emotion. [*E*_*si*_(*t*)- *E*_*ri*_(*t*)]* *R*_*sri*_ (*t*) represents the degree of emotional influence of *s* on *r*, which is not only based on the emotional difference between *s* and *r* but also related to the probability of emotional contagion between *s* and *r*. The greater the difference and the probability of infection, the larger the range of *r*’s emotional fluctuation. In this process, the emotional size of *s* does not change, mainly in terms of spreading emotions to others, and *s*’s existing emotions are not affected. Another possibility is that *r*, in addition to the positive range of emotional value, also plays the role of “*s*” after infection and then infects other associated individuals, accelerating the emotional contagion cycle.

Scenario three: *r* and *r*

7 $${E}_{r{1}i}(t+1)={d}_{r1i}(t)\ast {E}_{r1i}(t)+[{E}_{r1i}(t)-{E}_{r2i}(t)]\ast {R}_{r1r2i}(t)$$

8 $${E}_{r2i}(t+1)={d}_{r2i}(t)\ast {E}_{r2i}(t)+[{E}_{r2i}(t)-{E}_{r1i}(t)]\ast {R}_{r2r1i}(t)$$

When the adjacent nodes are all *r*, the emotional value of *r*_1_ at time *t* + 1 is the sum of the emotional value of *r*_1_ and the effective emotional influence of *r*_2_ and *r*_1_, and *r*_2_ is the same. *r* is a node infected by emotions. When *r* is in contact with another node, emotional contagion is a two-way process; however, the degree of mutual influence of the nodes may differ.

Scenario four: *m* and *s* or *r*

At time *t* + 1, when 0.33 < *E*_*mi*_ (*t* + 1) ≤ 0.66, the subject is in an immune state. Thus, when the value of *E*_*mi*_ (*t* + 1) is in the range (0.33, 0.66], the individual has been converted to *m* by default. However, when *E*_*m*_ (*t* + 1) ≤ 0.33 or *E*_*m*_ (*t* + 1) > 0.66, *m* moves to the immune state and is transformed into *s* or *r* when *s* has emotional influence on *m*. At time *t* + 1, when 0.33 < *E*_*ri*_ ≤ 0.66, the emotional state of the individual has not changed and is still in the immune state. When *E*_*ri*_ > 0.66, the subject’s emotion has been infected to a positive state (*r* + 1*, m*−1); when *E*_*ri*_ ≤ 0.33, the subject has been infected to a negative state (*r* + 1, *m*−1).

### Transformation of emotional contagion subjects

*s, r* and *m* can be transformed into each other under certain conditions. For example, at time *t*, *m*’s emotions are activated and converted to *s* with a probability *f*_*si*_ (*t*); thus, at time *t* + 1, the total number of *s* becomes the number of people at time *t* plus the number of people just converted from *m*, which is the same as *r*. The total number of *m* at time *t* + 1 becomes the number of people converted to *r* and *m* by the network number scale *N* minus the number converted to *r* and *m*. The calculation is as follows:

9 $${s}_{i}(t+1)={s}_{i}(t)+{f}_{si}(t)\ast {m}_{i}(t)$$

10 $${r}_{i}(t+1)={r}_{i}(t)+{f}_{ri}(t)\ast {m}_{i}(t)$$

11 $${m}_{i}(t+1)=N-{s}_{i}(t+1)-{r}_{i}(t+1)$$

12 $${f}_{si}(t)\ast {m}_{i}(t)+{f}_{ri}(t)\ast {m}_{i}(t)\le {m}_{i}$$

## Data Availability

All data generated or analyzed during this study are included in this published article.

## References

[1] Hatfield E, Cacioppo JT, Rapson RL (1992). Primitive emotional contagion. Emotion & Social Behavior.

[2] Barsade SG (2006). The ripple effect: Emotional contagion and its influence on group behavior. Administrative Science Quarterly.

[3] Stieglitz S, Dang XL (2013). Emotions and information diffusion in social media—sentiment of microblogs and sharing behavior. Journal of Management Information Systems.

[4] Coviello, L., *et al* Detecting emotional contagion in massive social networks. *Plos one* **9**, https://doi.org/journal.pone.0090315 (2014). 10.1371/journal.pone.0090315 PMC3951248 24621792

[5] Fan R, Zhao J, Chen Y, Xu K (2014). Anger is more influential than joy: Sentiment correlation in weibo. Plos One.

[6] Hatfield E, Cacioppo JT, Rapson RL (2010). Emotional contagion. Current Directions in Psychological Science.

[7] Verbeke W (1997). Individual differences in emotional contagion of salespersons: Its effect on performance and burnout. Psychology and Marketing.

[8] Mackie, D. M. & Hamilton, D. L. In *Affect, Cognition and Stereotyping* (eds Diane M., Mackie & David L., Hamilton) 371-383 (Academic Press, 1993)

[9] Iyer A, Leach CW (2008). Emotion in inter-group relations. European Review of Social Psychology.

[10] Zschaler G (2012). Adaptive-network models of collective dynamics. The European Physical Journal Special Topics.

[11] Gomes O (2015). Sentiment cycles in discrete-time homogeneous networks. Physica A: Statistical Mechanics and its Applications.

[12] Vosoughi S, Roy D, Aral S (2018). The spread of true and false news online. Science.

[13] Kwon KH, Bang CC, Egnoto M, Rao RH (2016). Social media rumors as improvised public opinion: Semantic network analyses of twitter discourses during Korean saber rattling 2013. Asian Journal of Communication.

[14] Ma J, Li D, Tian Z (2016). Rumor spreading in online social networks by considering the bipolar social reinforcement. Physica A: Statistical Mechanics and its Applications.

[15] Wang X, Zhang L, Lin Y, Zhao Y, Hu X (2016). Computational models and optimal control strategies for emotion contagion in the human population in emergencies. Knowledge-Based Systems.

[16] Na K, Garrett RK, Slater MD (2018). Rumor acceptance during public health crises: Testing the emotional congruence hypothesis. Journal of Health Communication.

[17] Michelson G, Mouly S (2000). Rumour and gossip in organisations: A conceptual study. Management Decision.

[18] Hatfield, E., Carpenter, M. & Rapson, R. L. In *Collective Emotions* 108–122 (Oxford University Press, 2014).

[19] Wrobel, M. & Imbir, K. Broadening the perspective on emotional contagion and emotional mimicry: The correction hypothesis. *Perspective on Psychological Science* **14**, 437–451 (2019). 10.1177/1745691618808523 30844340

[20] Bae Y, Lee H (2012). Sentiment analysis of twitter audiences: Measuring the positive or negative influence of popular Twitterers. Journal of the American Society for Information Science and Technology.

[21] Difonzo N (2013). Rumour research can douse digital wildfires. Nature.

[22] Berinsky, Adam J (2017). Rumors and health care reform: Experiments in political misinformation. British Journal of Political Science.

[23] Kuang W. New-media’s public opinions of the mass incident. *Sociology, Media and Journalism in China*. 225-253 (Springer,Singapore, 2018).

[24] Zeng, L., Starbird, K. & Spiro, E. S. Rumors at the speed of light? Modeling the rate of rumor transmission during crisis. *Hawaii International Conference on System Sciences (HICSS)*. IEEE. 1969–1978 (2016).

[25] Si XM, Wang WD, Zhai CQ, Ma Y (2016). A topic evolution model with sentiment and selective attention. Physica A Statistical Mechanics & Its Applications.

[26] Gosling SD, Augustine AA, Vazire S, Holtzman N, Gaddis S (2011). Manifestations of personality in online social networks: Self-reported Facebook-related behaviors and observable profile information. Cyberpsychology, Behavior, and Social Networking.

[27] Bordia P, DiFonzo N (2002). When social psychology became less social: Prasad and the history of rumor research. Asian Journal of Social Psychology.

[28] Borge-Holthoefer, J. & Moreno, Y. Absence of influential spreaders in rumor dynamics. *Physical Review E - Statistical, Nonlinear, and Soft Matter Physics* **85**, 10.1103/PhysRevE.85.026116 (2012). 10.1103/PhysRevE.85.026116 22463288

[29] Durupinar F, Gudukbay U, Aman A, Badler NI (2016). Psychological parameters for crowd simulation: from audiences to mobs. IEEE Trans Vis Comput Graph.

[30] Ferrara E, Yang Z (2015). Measuring emotional contagion in social media. PloS one.

[31] Wróbel, M. I can see that you’re happy but you’re not my friend: Relationship closeness and affect contagion. *Journal of Social and Personal Relationships* **35**, 1301-1318, https://doi.org/0265407517710820 (2017).

[32] Zhao L (2014). Sentiment contagion in complex networks. Physica A Statistical Mechanics & Its Applications.

[33] Fu L, Song W, Lv W, Lo S (2014). Simulation of emotional contagion using modified SIR model: A cellular automaton approach. Physica A: Statistical Mechanics and its Applications.

[34] Zhu H, Ma J (2019). Analysis of SHIR rumor propagation in random heterogeneous networks with dynamic friendships. Physica A: Statistical Mechanics and its Applications.

[35] Nekovee M, Moreno Y, Bianconi G, Marsili M (2007). Theory of rumour spreading in complex social networks. Physica A: Statistical Mechanics and its Applications.

[36] Li H, Sakamoto Y (2014). Social impacts in social media: An examination of perceived truthfulness and sharing of information. Computers in Human Behavior.

[37] Sahafizadeh E, Tork Ladani B (2018). The impact of group propagation on rumor spreading in mobile social networks. Physica A: Statistical Mechanics and its Applications.

[38] Bordia P, Difonzo N (2004). Problem solving in social interactions on the internet: Rumor as social cognition. Social Psychology Quarterly.

[39] Mcpherson M, Cook SLM (2001). Birds of a feather: Homophily in social networks. Annual Review of Sociology.

[40] Deng L, Liu Y, Zeng QA (2012). How information influences an individual opinion evolution. Physica A: Statistical Mechanics and its Applications.

[41] Bosse, T., Duell, R., Memon, Z. A., Treur, J. & Van Der Wal, C. N. A multi-agent model for emotion contagion spirals integrated within a supporting ambient agent model. *Principles of Practice in Multi-Agent Systems, 12th International Conference*. Proceedings. Springer-Verlag (2009).

[42] Xu K, Zhang S, Chen H, Li HT (2014). Measurement and analysis of online social networks. Chinese Journal of Computers.

[43] Berger J (2014). Word of mouth and interpersonal communication: A review and directions for future research. Journal of Consumer Psychology.

[44] Lorenzo C (2014). Detecting emotional contagion in massive social networks. Plos One.

[45] Paukert AL, Pettit JW, Amacker A (2008). The Role of Interdependence and Perceived Similarity in Depressed Affect Contagion. Behavior Therapy.

[46] Trevors GJ, Muis KR, Pekrun R, Sinatra GM, Winne PH (2016). Identity and Epistemic Emotions During Knowledge Revision: A Potential Account for the Backfire Effect. Discourse Processes.

[47] Ozturk, P., Li, H. & Sakamoto, Y. Combating rumor spread on social media: The effectiveness of refutation and warning. *Hawaii International Conference on System Sciences* 2015-March, 2406–2414 (IEEE Computer Society, 2015).

[48] Zhu H, Kong Y, Wei J, Ma J (2018). Effect of users’ opinion evolution on information diffusion in online social networks. Physica A: Statistical Mechanics and its Applications.

[49] Si XM, Wang WD, Ma Y (2016). Role of propagation thresholds in sentiment-based model of opinion evolution with information diffusion. Physica A: Statistical Mechanics and its Applications.

[50] Başak AE, Güdükbay U, Durupınar F (2018). Using real life incidents for creating realistic virtual crowds with data-driven emotion contagion. Computers & Graphics.

[51] Dezecache G, Jacob P, Gre′zes J (2015). Emotional contagion: Its scope and limits. Trends in Cognitive Sciences.

[52] Warren ZJ, Power SA (2015). It’s contagious: Rethinking a metaphor dialogically. *Culture &*. Psychology.

[53] Neumann R, Strack F (2000). “Mood contagion”: The automatic transfer of mood between persons. Journal of Personality and Social Psychology.

[54] Hess U, Fischer A (2014). Emotional Mimicry: Why and when we mimic emotions. Social and Personality Psychology Compass.

[55] Schul Y, Mayo R, Burnstein E (2008). The value of distrust. Journal of Experimental Social Psychology.

[56] Lobato E, Mendoza J, Sims V, Chin M (2014). Examining the relationship between conspiracy theories, paranormal beliefs, and pseudoscience acceptance among a university population. Applied Cognitive Psychology.

[57] Difonzo N (2010). Ferreting facts or fashioning fallacies? factors in rumor accuracy. Social & Personality Psychology Compass.

[58] DiFonzo N, Bordia P (2007). Rumor, gossip and urban legends. Diogenes.

[59] Bordia P, Difonzo N, Schulz CA (2000). Source characteristics in denying rumors of organizational closure: Honesty is the best policy. Journal of Applied Social Psychology.

[60] Berinsky, Adam J (2017). Rumors and health care reform: Experiments in political smisinformation. British Journal of Political Science.

[61] Huo LA, Cheng Y, Liu C, Ding F (2018). Dynamic analysis of rumor spreading model for considering active network nodes and nonlinear spreading rate. Physica A: Statistical Mechanics and its Applications.

[62] Liu W, Wu X, Yang W, Zhu X, Zhong S (2019). Modeling cyber rumor spreading over mobile social networks: A compartment approach. Applied Mathematics and Computation.

[63] Liu, Y., Tang, M., Zhou, T. & Younghae, D. Core-like groups result in invalidation of identifying super-spreader by k-shell decomposition. *Scientific Reports* **5** (2015). 10.1038/srep09602 PMC5386204 25946319

[64] Brockmann D, Helbing D (2013). The hidden geometry of complex, network-driven contagion phenomena. Science.

[65] Daley DJ, Kendall DG (1965). Stochastic rumours. Ima Journal of Applied Mathematics.

[66] Doer B, Fouz M, Friedrich T (2012). Why rumors spread so quickly in social networks. Communications of the ACM.

